# CD14^+^ monocytes: the immune communication hub in early vasculitis symptoms of Kawasaki disease

**DOI:** 10.3389/fimmu.2025.1557231

**Published:** 2025-03-26

**Authors:** Sirui Song, Liqin Chen, Yuanyuan Zhou, Yanbing Xu, Guang Li, Libing Shen, Tingting Xiao, Min Huang

**Affiliations:** ^1^ Department of Cardiology, Shanghai Children’s Hospital, School of Medicine, Shanghai Jiao Tong University, Shanghai, China; ^2^ Institute of Pediatric Infection, Immunity, and Critical Care Medicine, Shanghai Jiao Tong University School of Medicine, Shanghai, China; ^3^ Daozhi Precision Medicine Technology (Shanghai) Co., Ltd, Shanghai, China; ^4^ International Human Phenome Institutes (Shanghai), China, Shanghai, China

**Keywords:** CD14 + monocytes, Kawasaki disease, single-cell sequencing, cell communication, WGCNA (weighted gene co- expression network analyses)

## Abstract

**Background:**

Kawasaki Disease (KD) is an acute systemic vasculitis syndrome predominantly affecting children, with a propensity to induce coronary artery lesions. Aberrant immune activation and cytokines cascade reactions are involved in its pathogenesis. The aim of this study is to investigate the changes in immune cell communication during the course of KD and to identify potential biomarkers.

**Methods:**

The study enrolled seven pediatric patients diagnosed with Kawasaki Disease (KD) between December 2019 and December 2021. Single-cell RNA sequencing (scRNA-seq) technology was utilized to analyze peripheral blood mononuclear cells (PBMCs). Bioinformatics methods including quality control, dimensionality reduction, cell annotation, differential expression analysis, cell communication analysis, and co-expression network analysis were employed for data processing and analysis.

**Results:**

This study utilized single-cell sequencing technology to uncover the dynamics of immune cell communication during the course of KD, revealing a significant increase in the number of CD14^+^ monocytes in the early stages of vasculitis, which play a central role in cell-cell communication. SELPLG was identified as a particularly crucial gene in the signal transduction among immune cells. The study also observed various cellular communication patterns of vasculitis at different time points and identified co-expression modules related to ribosomal function, cell proliferation, and immune responses in CD19^+^ B cells, CD4^+^ T cells, CD8^+^ T cells, CD14^+^ monocytes, and CD16^+^ monocytes. Notably, the expression of the ITK gene in CD14^+^ monocytes stood out. Furthermore, MHC-I genes were the most active molecules involved in signal transduction, and the expression of CD40 genes increased with the prolongation of vasculitis duration.

**Conclusion:**

CD14^+^ monocytes play a pivotal role in cellular communication during the activation process of KD vasculitis, with SELPLG and ITK as important communication signal genes. These findings provide a novel perspective for the discovery of biomarkers, prediction of disease progression, and the development of targeted treatment strategies for KD.

**Clinical Trial Registration:**

http://www.medresman.org.cn/pub/cn/proj/projectshow.aspx?proj=7739, identifier ChiCTR, ChiCTR2100044729.

## 1 Background

Kawasaki Disease (KD) is an acute systemic vasculitis syndrome predominantly affecting children, characterized by the involvement of small and medium-sized arteries, particularly the coronary arteries, and marked by the activation of the immune system and widespread damage to the vascular endothelium (1, 2). Although the precise etiology remains to be fully elucidated, some studies have indicated that aberrant immune activation and a cascade of cytokine reactions are involved in the pathogenesis of KD (3). Many researchers propose that KD may be a vasculitis reaction triggered by an infection-induced superantigen that activates autoimmunity (4, 5). A critical question arises regarding the extent of immune cell activation in KD patients when vasculitis symptoms appear post-infection. The communication among immune cells coordinates innate and adaptive immune responses, involving the activation and interaction of neutrophils, monocytes, T cells, B cells, and others (6). These cells amplify or modulate the inflammatory response by secreting cytokines and chemokines, thereby affecting the severity of the disease and the risk of cardiovascular complications. A thorough understanding of these communication processes can aid the discovery of biomarkers, predict disease progression, and provide a scientific basis for the development of targeted treatment strategies (7).

In the realm of inflammatory response dynamics, the application of single-cell RNA sequencing (scRNA-seq) technology has unveiled alterations in multiple immune cell subsets during the acute phase of KD. Wang et al. previously delineated a peripheral blood mononuclear cell (PBMC) landscape in KD patients, which included a reduction in the numbers of immature CD8^+^ T cells, helper T cells, and B cells, alongside an increase in the numbers of major immune-related T cells and natural killer T (NKT) cells (8). These variations at the single-cell level reflect immune cell dysregulation in KD, suggesting that disease-specific immune cell subsets may play a pivotal role in the pathogenesis of KD (1). Moreover, functional enrichment analysis revealed that cell activation, lymphocyte activation, and immune system process regulation are three key biological functions shared by all four subsets of T cells and B cells. scRNA-seq analysis also identified aberrantly activated signaling pathways, such as the mammalian target of rapamycin (mTOR) pathway and others associated with bacterial or viral infections, offering potential molecular targets for therapeutic intervention (9). Chen et al. by comparing single-cell data from KD and COVID-19 patients, discovered similarities in the neutrophil activation and reduced expression of MHC class II molecules between the two diseases, which may indicate shared pathophysiological mechanisms (10).

The application of single-cell RNA sequencing (scRNA-seq) technology has previously enhanced our understanding of immune cell changes and heterogeneity in KD. However, to date, no study has focused on the communication among immune cells and the co-expression module analysis of KD immune cells derived from single-cell sequencing results. The objective of this study is to investigate the alterations in the peripheral blood immune cell landscape of Kawasaki Disease (KD) at multiple time points, with a particular focus on the duration of vasculitis as a reference. The study aims to ascertain the relationship between the communication patterns of immune cells and the chronicity of vasculitis, as well as to characterize the dynamics of immune co-expression networks at various stages of the disease. Additionally, this research endeavors to uncover the interplay between cell-specific co-expression modules and extracellular signaling molecules.

## 2 Materials and methods

### 2.1 Patients

Seven pediatric patients diagnosed with Kawasaki Disease (KD) and admitted to the cardiology department between December 2019 and December 2021 were selected as the subjects of this study, All KD patients met the classic KD diagnostic criteria established by the American Heart Association (AHA) in 2017 (11), which include fever of unknown origin and at least four of the five principal clinical features (conjunctivitis, oral changes, limb changes, rash, and cervical lymphadenopathy). Patients fulfilling these criteria were diagnosed as complete KD. To elucidate the relationship between gene expression profiles and the clinical manifestations of KD patients, this study introduced a novel approach, utilizing the duration of vasculitis symptoms for patient stratification rather than the traditional reliance on fever duration. The early vasculitis group was defined as those with KD where the duration of all relevant vasculitis symptoms at diagnosis was less than 3 days. The late vasculitis group was defined as those with KD where the duration of any vasculitis symptom at diagnosis was 3 days or more. All patients were administered a high dose of intravenous immunoglobulin (IVIG) at 2g/kg in conjunction with oral aspirin at 30mg/kg/day following the diagnosis of KD. Blood samples were collected at the same time point in the course of the disease for all KD patients, with pre-treatment specimens collected on the day of diagnosis and prior to IVIG treatment. Healthy controls were recruited from outpatient health examinations, including individuals under the age of six with no recent history of fever, infection, or vaccination. Children with a history of KD and those with autoimmune diseases were also excluded from the control group. Common febrile children hospitalized during the same period were selected as the fever control group. Patients meeting the following criteria were considered fever control individuals: ① confirmed infection, including bacterial meningitis, bacterial pneumonia, influenza, urinary tract infections, etc.; ② fever for at least 3 days; ③ aged between 1 month and 5 years. Control group children underwent single-cell sequencing only once at the time of recruitment. The analysis included seven samples, comprising a re-analysis of previously published data from our group (8), supplemented with three new samples. Experimental samples were derived from the remaining blood after testing, with informed consent obtained. This study has been reviewed and approved by the Ethics Committee of Shanghai Children’s Hospital.

### 2.2 Single-cells preparation and sequencing

Venous blood samples of 2ml were collected from each donor using EDTA-coated tubes. Fresh blood was immediately processed for the preparation of peripheral blood mononuclear cell (PBMC) suspensions. The criteria for the cell suspensions were as follows: greater than 85% cell viability, a total cell count exceeding 200,000 cells, and cell diameters ranging from 7 to 60 µm. The cell suspensions were free of red blood cells, absence of cell clumping, and no significant cellular debris or aggregates. PBMC suspensions were subjected to single-cell library construction within 30 minutes. The target cell number for each sample was set at 1,000 to 10,000, with a sequencing depth of 30M bases per cell for 5’ gene expression and 3M bases per cell for VDJ sequencing.

### 2.3 Bioinformatics analysis

Data Quality Control: Stringent quality control measures were employed across all samples, with the following criteria: the number of detected unique RNA molecules (“n_featureRNA”) was maintained within the range of 200 to 2500, serving as an indicator of sequencing saturation, cell size, and complexity; the percentage of mitochondrial genes was set to be below 5%.

Dimensionality Reduction: The count matrix was log-normalized with a scaling factor of 10,000, and the 2,000 most variable genes were identified for further dimensionality reduction. The integrated matrix was scaled, and the principal components from principal component analysis (PCA) were utilized for Uniform Manifold Approximation and Projection (UMAP). The optimal number of PCA dimensions for downstream clustering and visualization was determined using the ElbowPlot in Seurat. Cluster identification was performed using the FindClusters function in Seurat, applying a shared nearest neighbor (SNN) graph-based clustering algorithm on the PCA-reduced data, with dims set to 1:50 and a resolution of 0.5. UMAP was employed to visualize the cells in a two-dimensional space (12, 13).

Cell Annotation: Cell identities were initially determined using SingleR (version 2.4.1) (14), a software tool that compares the transcriptome of each cell population with various reference datasets, including the Human Primary Cell Atlas, Blueprint/ENCODE, Immune Cell Expression Database, Novershtern Hematopoietic Data, and Monaco Immune Data. Due to some inconsistencies and ambiguities in the automatic assignments, the FindMarkers function in Seurat was utilized to identify marker genes for each cluster. Subsequently, cell cluster annotations were manually refined based on these marker genes, along with the latest single-cell research findings and the Cell Type Annotation (ACT) web server (http://xteam.xbio.top/ACT/index.jsp).

Differential Expression Analysis: Subsequently, DESeq2 (version 1.28.1) was employed to analyze differential expression under various conditions, estimating the variance-mean dependence of count data and testing for differential expression based on the negative binomial distribution (15). ANOVA (Analysis of Variance) is employed to assess the differences in cell numbers across various groups.

Cell Communication Analysis: To investigate ligand-receptor complex-mediated cell-cell interactions (CCI), especially between natural killer (NK) cells and other subsets within single-cell transcriptomic data, the SingleCellSignalR tool was utilized. This tool predicts interactions between ligands (L) and receptors (R) across different cell types. The LRscore is calculated as the product of the average expression of ligands in one cell type and the average expression of receptors in another, serving as a proportionality index. Dimensionality reduction was applied to ensure that the total cell counts between the two groups were comparable across samples before CCI analysis. Heatmaps were generated to visualize interactions with LRscores exceeding 0.5 between NK cells and other cell types within each sample, with missing LRscores imputed as 0. The average number of interactions per sample site was also calculated. Statistically significant differences in cell interactions were identified using Wilcoxon tests with a p-value threshold set at 0.05. Subsequent analysis of specific interactions was performed using the same non-parametric test by constructing CellChat objects and calculating the similarity matrix between the pathways (16). Isolated pathways were successfully identified, that are, those pathways with a similarity value of 0 to all other pathways.

Co-expression Network Analysis:

In the context of co-expression network analysis, given that single-cell sequencing expression matrices are inherently sparse, and considering the sensitivity of Weighted Correlation Network Analysis (WGCNA) to data sparsity, the WGCNA (R package version 1.71) was applied to the constructed meta-cells for subsequent analysis (17). The “WGCNA” package identified hub genes that were significantly associated with the CAF score. Initially, the top 5000 variable expression profiles from the cohort were used as input. A soft threshold was established to cluster an adjacency matrix and to identify a hub module. The strongest positive correlation coefficient between the module and the samples and their corresponding soft threshold was selected for further analysis by calculating the Pearson correlation coefficients. Subsequently, the gene significance (GS) and module membership (MM) for each gene within the hub module were measured. Ultimately, genes within the module were filtered as potential CAF-associated genes using a threshold of MM > 0.6 and GS > 0.6.

Gene Ontology (GO) Analysis: The Gene Ontology (GO) database was utilized to functionally annotate the differentially expressed genes (DEGs), with the aim of identifying significantly enriched biological processes (BP), molecular functions (MF), and cellular components (CC). The enrichment significance was assessed using a statistical test based on the hypergeometric distribution.

UpSet Analysis: To illustrate the shared and unique elements among different expression modules, UpSet analysis was employed. Utilizing the UpSetR package in R, data were formatted through the fromList or fromExpression functions. Subsequently, the UpSet diagram was plotted with the upset function, adjusting parameters for sorting, displaying the number of sets, and proportions.

## 3 Results

### 3.1 CD14^+^ monocytes significantly increased in the early stages of vasculitis

#### 3.1.1 General situation

All children participating in this study are depicted in **Table 1**. Seven Kawasaki Disease (KD) patients were involved in the single-cell sequencing process, consisting of four males and three females with an average age of 3.0 ± 1.4 years (range, 1.6-5.3 years). The early vasculitis group comprised three children, while the late vasculitis group included four children. All patients responded to IVIG treatment and did not develop coronary artery aneurysms (CALs). A control group of three children of similar age was selected, which included two healthy children and one child with a common respiratory infection accompanied by fever. The control group consisted of one boy and two girls, aged between 1.1 and 5.3 years. **Table 1** provides detailed information about the children in each group.

**Table 1:** The demographic and clinical characteristics of children in three groups.

Group	Early vasculitis group			Late vasculitis group				Controls		
Patients	P1	P2	P3	P4	P5	P6	P7	C1	C2	C3
Gender	Male	Male	Male	Female	Female	Female	Male	Male	Female	Female
Age (years)	2.1	2.0	4.7	1.6	5.3	3.3	1.9	1.1	5.3	4.9
Duration of fever (days)	4	6	4	3	5	6	6	\	\	4
Congestion of the bulbar conjunctiva (days)	1	1	2	3	5	2	5	\	\	\
changes of lips and oral cavity (days)	1	1	1	3	1	4	5	\	\	\
Rash and erythema multiforme (days)	2	2	2	3	4	1	6	\	\	\
Swelling of the extremities (days)	1	1	1	3	\	\	\	\	\	\
Perianal flushing and desquamation (days)	\	1	1	1	\	1	1	\	\	\
Enlargement of cervical lymph nodes (days)	1	1	1	1	1	1	1	\	\	\

P, patient; C, control; C3 was diagnosed with pneumonia.

### 3.1.2 Dynamic changes in the main cell subsets of PBMCs in KD peripheral blood, classified by the duration of vasculitis

**Figure 1:**
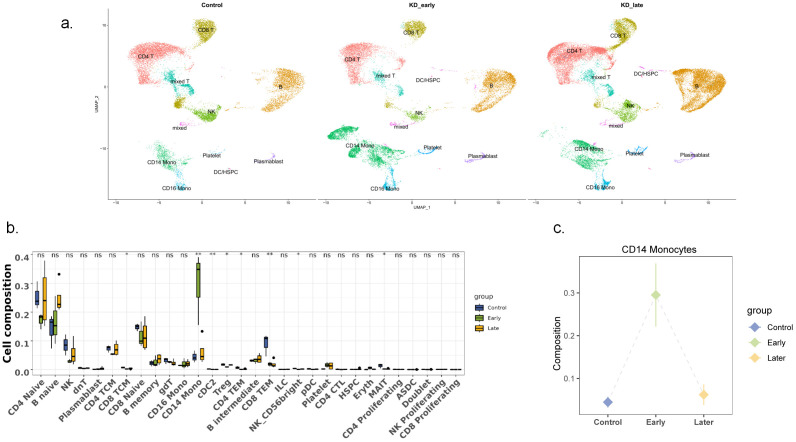
Single-cell landscape of PBMCs in control, KD early and KD late patients. **(a)** Two-dimensional representation of cell types identified by scRNA-seq: The dimensional reduction is performed with the uniform manifold approximation and projection (UMAP). Each dot represents a cell, which is colored according to cell type. The cells are pooled across all patients and separated by conditions: control group, KD Early vasculitis group,KD Late vasculitis group. The type of each condition is shown in the figure. **(b)** Comparison of major cell types across conditions: Percentage of each cell type in PBMCs revealed by scRNA-seq. The proportion of each immune cell was compared among the early vasculitis group and the late vasculitis group of KD and the control group. **(c)** CD14+ monocytes increased significantly in early vasculitis group. ns P>0.5, * P<0.5, ** P<0.05.

Single-cell RNA sequencing (scRNA-seq) was conducted on peripheral blood mononuclear cells (PBMCs) extracted from samples using the 10× Genomics platform. Approximately 12,000 PBMCs from each sample were loaded onto the platform, and after sequencing, 6,000 cells were recovered. A total of 48,761 cells passed quality control, with 34,073 cells from KD patients before treatment and 14,688 cells from the control group. PBMCs were summarized based on conditions, revealing differences in gene expression between KD patients (categorized by the duration of vasculitis) and the control group (**Figure 1**). The main cell types of PBMCs were identified, including T cells, natural killer (NK) cells, B cells, monocytes, myeloid dendritic cells, plasmacytoid dendritic cells, and hematopoietic stem/progenitor cells (HSPCS). A few residual red blood cells and megakaryocytes were also mixed in the PBMCs (**Figure 1**). These included CD4^+^ T cells (28.7%), CD8^+^ T cells (16.6%), natural killer (NK) cells (6.2%), CD19^+^B cells (26.7%), monocytes (14.5%), myeloid dendritic cells (mDCs) (<0.1%), plasmacytoid dendritic cells (pDCs) (<0.1%), and hematopoietic stem/progenitor cells (HSPCs) (<0.1%). When classified by the duration of vasculitis, among the more abundant cells, CD19^+^ B lymphocytes and their subsets increased in number with the prolongation of vasculitis symptoms; CD4^+^ T lymphocytes and CD8^+^ T lymphocytes decreased in number in the early stages of vasculitis and increased in the later stages. None of these differences were significant. Notably, CD14^+^ monocytes significantly increased in the early stages of vasculitis (P<0.05) (**Figure 1**).In the control group, the cell distribution comparison between healthy children and febrile children is presented in **Supplementary Figure 1**, where no significant differences were observed in the number of cells across various categories.

### 3.2 Different time points of vasculitis exhibit distinct cellular communication patterns, with CD14 monocytes playing a pivotal role in communication during the early stages of vasculitis

**Figure 2:**
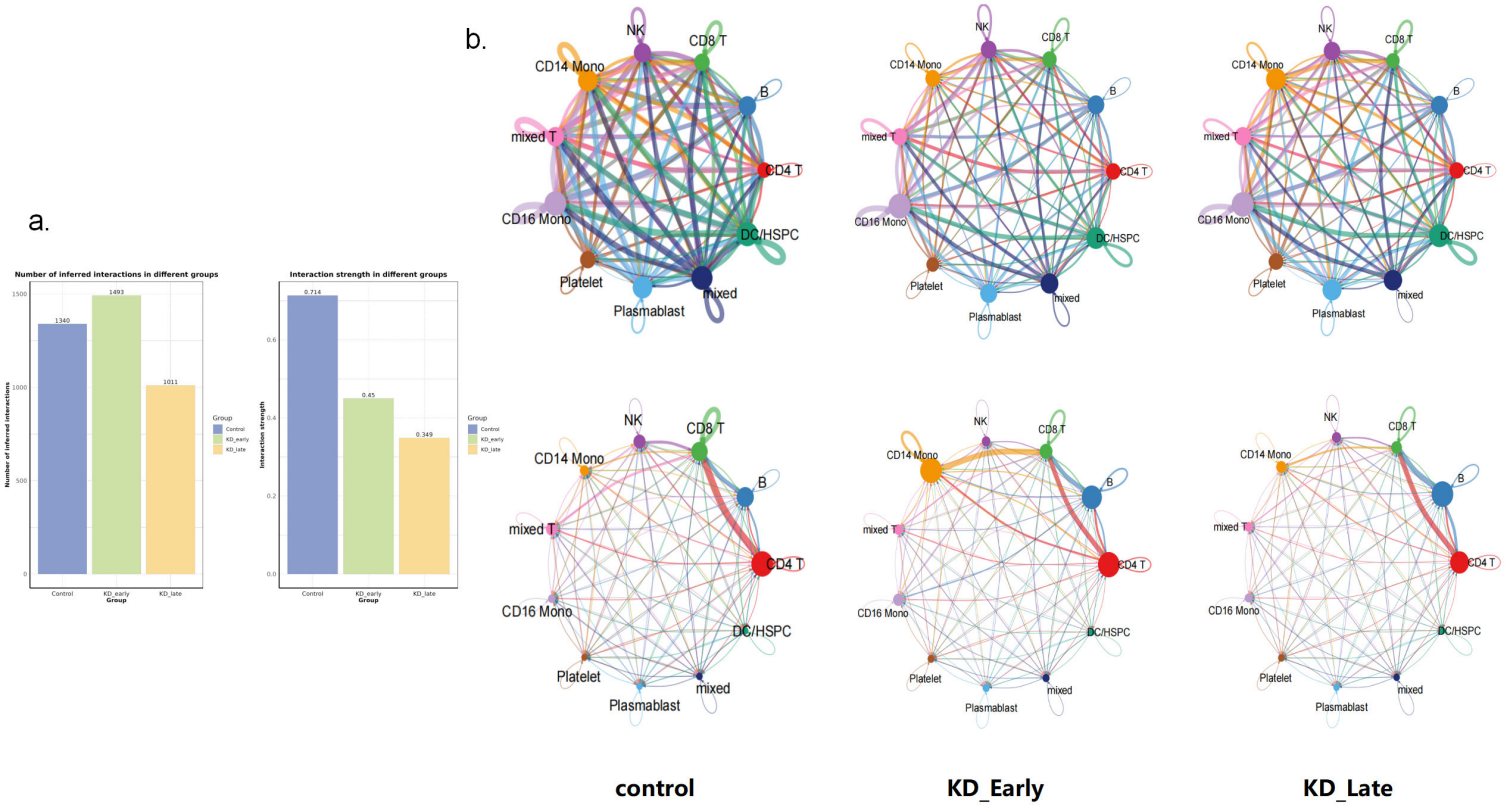
Cell-to-cell communication analyses for control, KD early and KD late patients. **(a)** The signal quantity and signal intensity of PBMCs in each group. **(b)** The network diagram showed the signal quantity and signal intensity of PBMCs in each group. Nodes represent cell types, edges represent interactions, and the thickness of the edges indicates the quantity of interactions in the upper figure, while in the lower figure it represents the strength of interactions.

**Figure 3:**
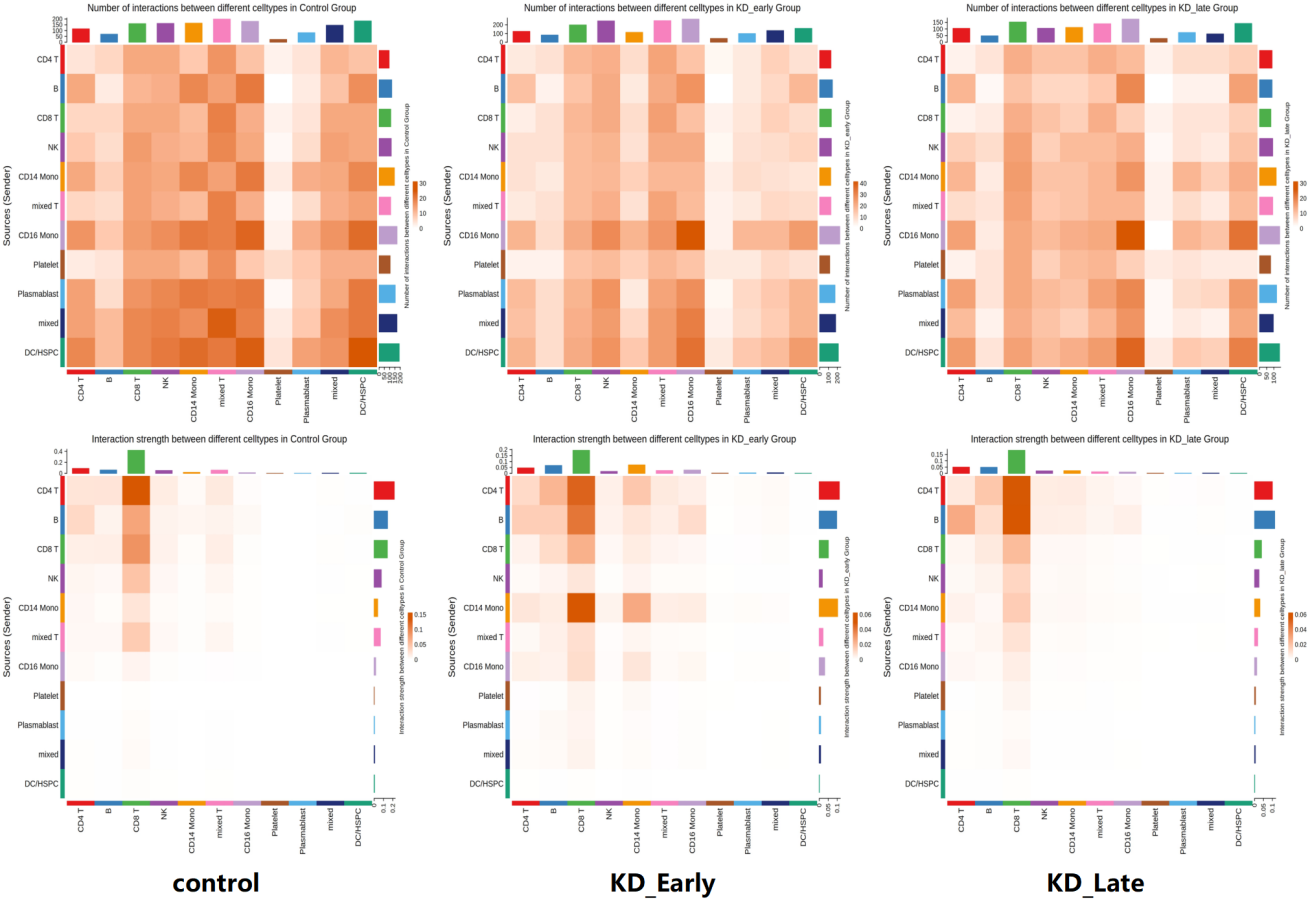
The communication intensity of PBMCs and the receiving and output signal intensity of different cells in each group. The intensity of colors in the heatmap corresponds to the quantity of signaling interactions between different cells in the upper figure, and to the strength of different signaling interactions in the lower figure.

Overall, as the duration of vasculitis extended, the number and intensity of cell-to-cell communications in the early stages of vasculitis showed a trend of increasing and then decreasing (**Figure 2a**). Under normal conditions, the number of cell communications among various immune cells was relatively balanced, with signal intensity mainly concentrated between CD8^+^ T cells and themselves (13), CD4^+^ T cells (6), CD19^+^ B lymphocytes (6), and NK cells (13), especially the interaction signals between T lymphocytes were very strong. In the early stages of vasculitis, the number of cell communications increased between CD16^+^ monocytes and themselves and other cells. While cell communication intensity increased between CD8^+^ T cells and themselves (0.009) and CD4^+^ T cells (0.013), CD19^+^ B lymphocytes (0.013), a more significant change was observed in the communication between CD8 T^+^ cells and CD14^+^ monocytes. In the later stages of vasculitis, the overall number of cell communications decreased, and the proportion of communications between CD16^+^ cells and themselves and other various cells remained high. In terms of communication signal intensity, CD8+ T cells and CD4^+^ T cells and CD19^+^B cells dominated the communication intensity (**Figures 2b**, **3**).

**Figure 4:**
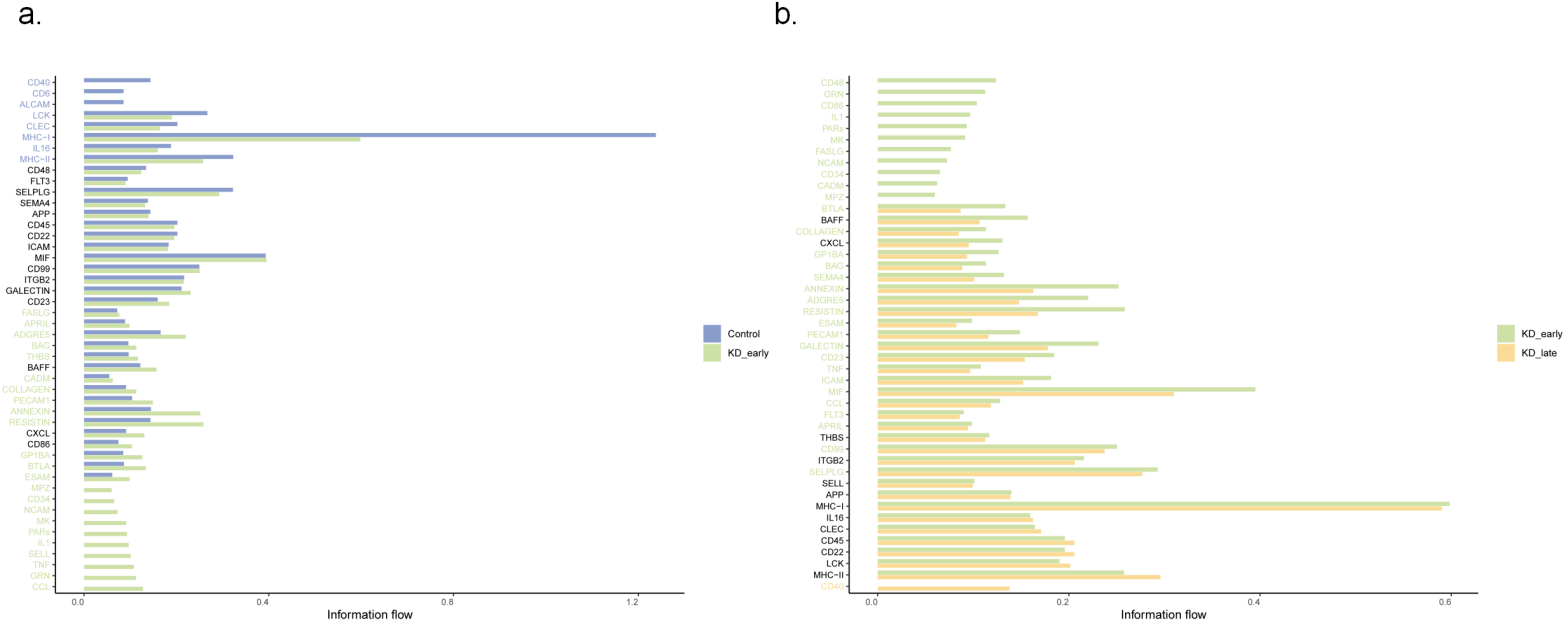
The comparative analysis of relative signal flow intensities between different biological markers at three distinct time points: **(a)** control versus KD_early and **(b)** KD_early versus KD_late. The horizontal axis represents the relative signal flow, ranging from 0.00 to 1.00, while the vertical axis lists various signaling molecules.

Using the method of identifying isolated pathways, two isolated pathways were recognized in the early stages of Kawasaki disease vasculitis: the PARs pathway and the CD34 pathway. These pathways exhibit similar gene functions and do not form significant connections with other pathways within the network. Notably, the difference in MHC-I gene expression was the most significant (P < 0.05). In the later stages of Kawasaki disease vasculitis, four isolated pathways were identified: PARS, FASLG, NCAM, and the CD34 signaling pathway. Among these, CD40 gene expression increased with the duration of vasculitis (P < 0.05) (**Figure 4**, **Supplementary Figure 2**).

**Figure 5:**
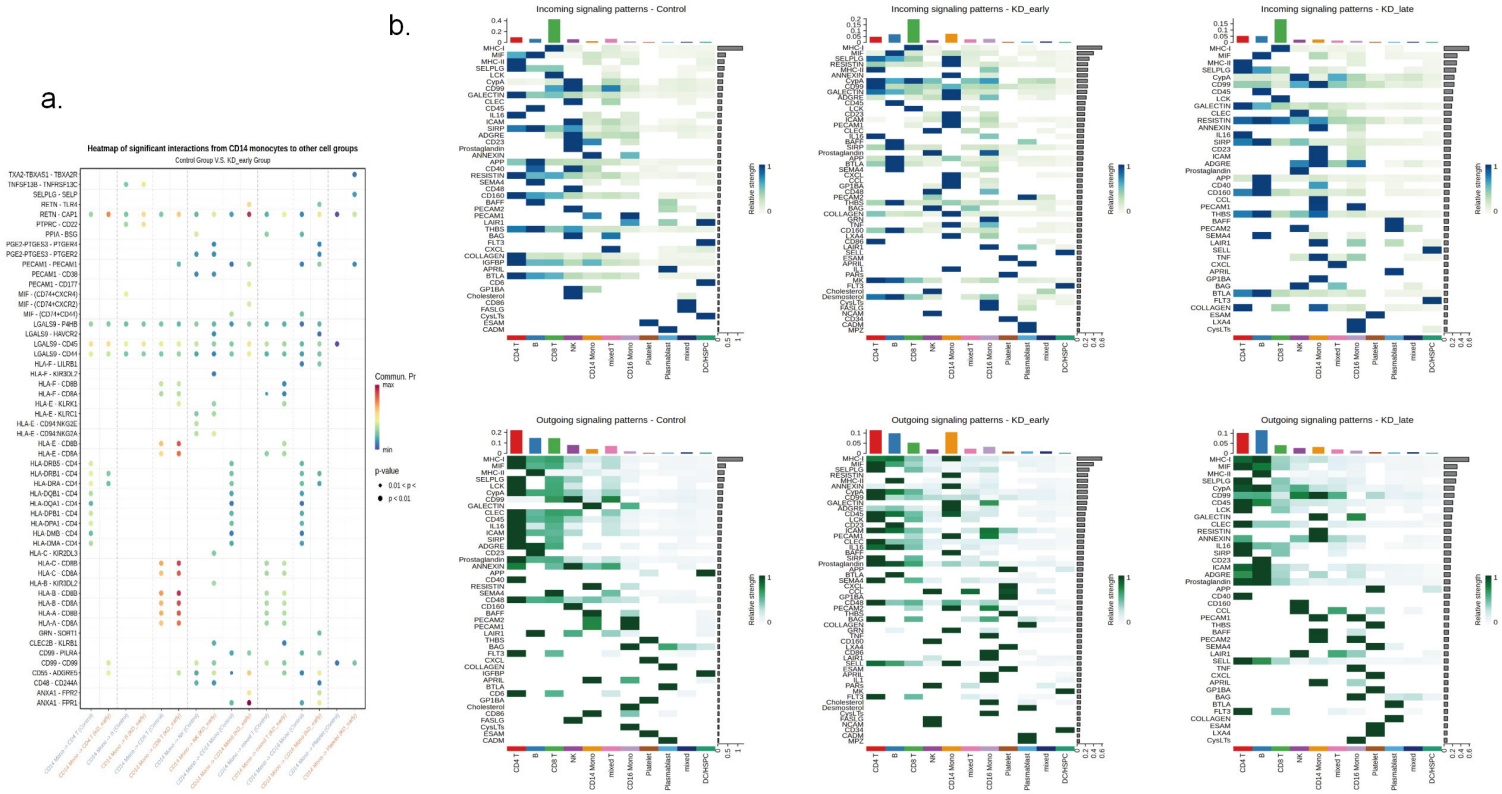
The expression patterns of signaling molecules in control, KD early and KD late patients. **(a)** The heatmap illustrated the expression quantity and intensity of signaling molecules involved in intercellular communication across different groups. The color intensity in the heatmap corresponds to the level of signaling molecule expression, providing a visual representation of the communication dynamics between various cellular populations. **(b)** The expression intensity of signaling molecules in communication between CD14 and different cells.

It was observed that regardless of the time period, CD8^+^ T cells recognized the most signals. Before vasculitis occurred, the signal molecules secreted by CD4^+^ T cells and B cells were mainly recognized by CD8^+^ T cells, among which the most obvious were MHC-I genes, mainly secreted by CD4^+^ T cells and recognized by receptors on CD8^+^ T cells, which is consistent with the normal antigen presentation and activation of T cell function by MHC-I molecules; in the early stages of vasculitis, CD14^+^ monocytes released the most signals, MHC-I genes were mainly secreted by CD14^+^ monocytes, and in the later stages of vasculitis, signals released by CD19^+^ B cells dominated, and MHC-I genes were also mainly released by CD19^+^ B cells, with peptides on the surfaces of these cells recognized and initiating an immune response by CD8^+^ T cells. However, there was no difference in the secretion and recognition of MHC-II genes between groups, all secreted by CD19^+^ B cells and recognized by CD4^+^ T cells (**Figure 5a**).

**Figure 6:**
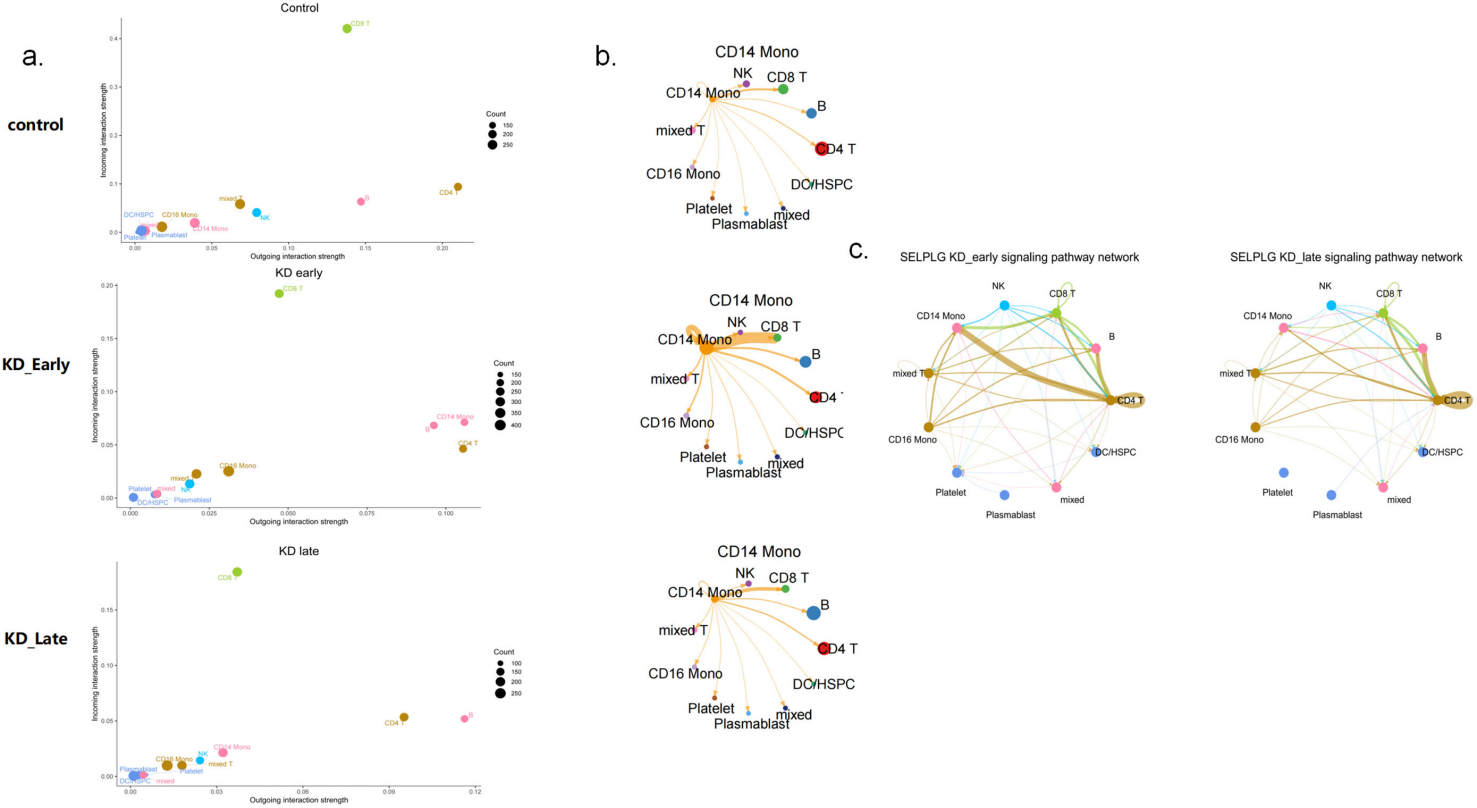
The signaling patterns and molecules of CD14 monocytes in control, KD early and KD late patients**. (a)** The strength of intercellular communication within the immune system, detailed the outgoing and incoming interaction strengths among various types of immune cells. The heatmap illustrates the intensity of these interactions, with a scale ranging from 0.00 to 0.15. **(b)** This figure highlights the CD14 monocyte subset, demonstrating its connections with various immune cells. The thickness of the edges reflects the strength of signaling between CD14 monocytes and different types of immune cells, with nodes representing the various cells. **(c)** The impact of SELPLG gene on the early and late signaling pathway networks in KD vasculitis. Nodes in the diagram represent various cell types, while edges denote the interactions between them. The thickness of the edges indicates the strength of SELPLG gene signaling among different immune cell types.

It can be seen that in the early stages of vasculitis, considering both the release and reception of signals, CD14^+^ monocytes occupy a central position in communication network. Among them, CD14^+^ monocytes communicate most closely with CD8^+^ T cells. An increase in the expression of cell surface HLA genes can be observed, presenting to CD8^+^ T cells and triggering an immune response (**Figure 5b**). Considering all differential inputs and outputs of signals, the SELPLG gene is the strongest gene in terms of signals output and input by CD14^+^ monocytes in the early stages of vasculitis (**Figure 6**).

### 3.3 Cellular co-expression network and GO analysis

Co-expression network analysis was conducted on cells that mainly receive and recognize signals, including CD19^+^ B lymphocytes, CD4^+^ T cells, CD8^+^ T cells, CD14^+^ monocytes, and CD16^+^ monocytes. The expression profiles of the top 5000 most highly expressed genes in each cell type were analyzed, and dynamic module identification was conducted across the three groups. This section aims to identify genes within co-expression networks for each cell type, revealing potential functional modules that DEG analysis alone cannot detect.

#### 3.3.1 CD19^+^ B cells

Using WGCNA analysis, The research identified distinct co-expression modules within B cells across three different groups. In the control group, three modules were found, with genes clustering in T cell activation (37 genes) and immune response activation (42 genes), particularly in NK cells. In contrast, vasculitis groups exhibited fewer modules, with genes primarily clustering in ribosomal subunit assembly and function, suggesting a shift towards cell proliferation and maintenance during disease progression (**Supplementary Figure 3**, **Supplementary Tables 1**, **2**).

#### 3.3.2 CD4^+^ T cells

The control group had one module with 91 genes, while the early vasculitis group had two modules (116 and 207 genes). The late vasculitis group had a single module with 144 genes. These modules highlight the dynamic nature of gene expression during disease progression, with early stages showing more complex expression patterns (**Supplementary Figure 4**, **Supplementary Tables 3**, **4**).

#### 3.3.3 CD8^+^ T cells

In the control group, a single module with 235 genes focused on ribosomal function was identified. The early vasculitis group had four modules, including one with 126 genes related to TH17 cell differentiation and two modules (40 and 196 genes) involved in leukocyte chemotaxis. The late vasculitis group had two non-specific modules (60 and 118 genes) related to ribosomal function. These findings suggest a shift from immune cell recruitment to general cellular maintenance in later stages (**Supplementary Figure 5**, **Supplementary Tables 5**, **6**).

#### 3.3.4 CD14^+^ monocytes

**Figure 7:**
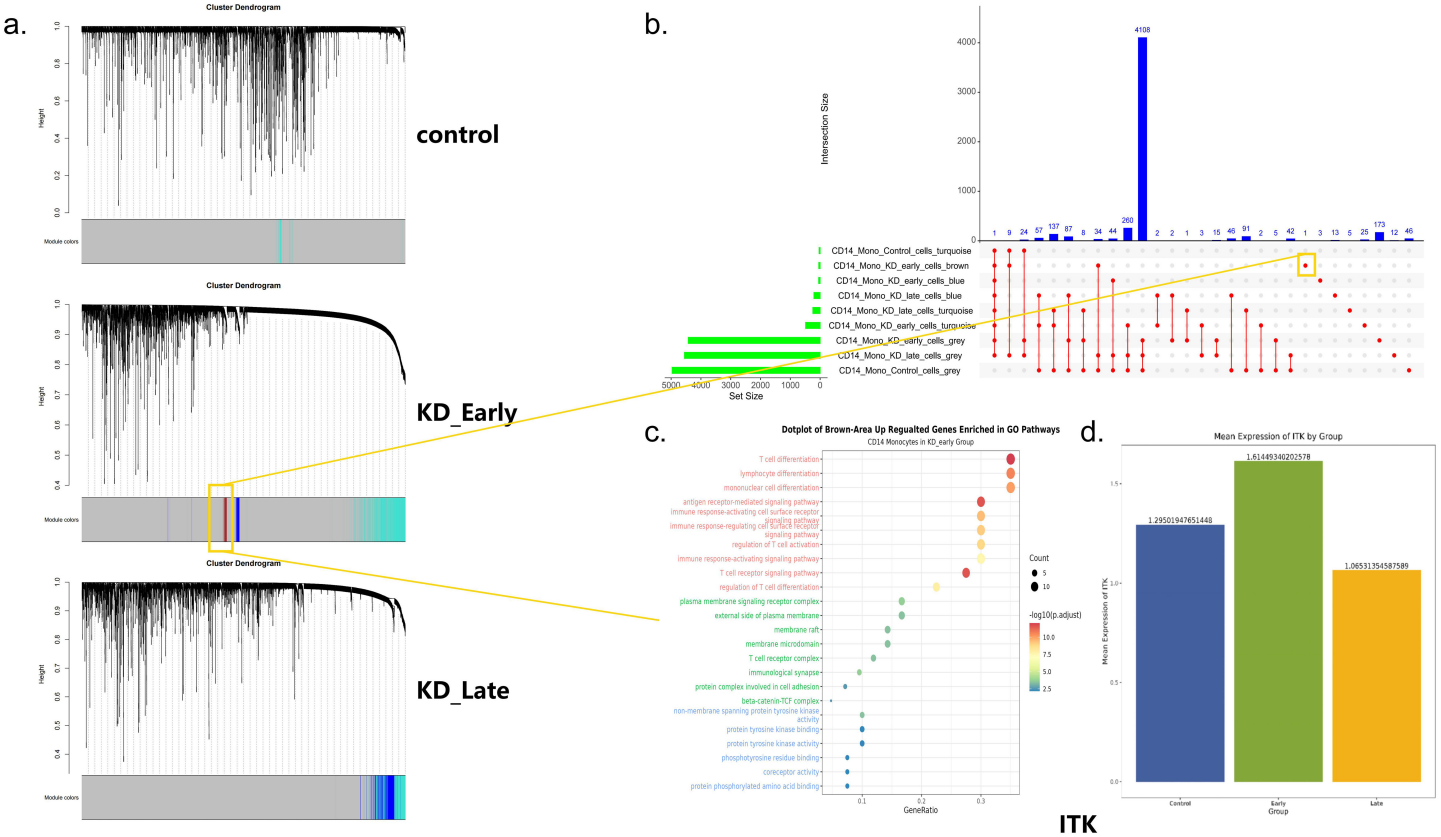
**(a)** A dendrogram constructed using the “average” linkage strategy. The height of the dendrogram represents the merging distance between different clusters, while different colors are used to distinguish between various cluster modules. The vertical axis of the plot displays the height of the clusters, and the horizontal axis lists the data points or objects involved in the clustering. **(b)** The UpSet plot illustrates the intersection sizes among various datasets, each represented by different colors in the dendrogram. The horizontal axis denotes the intersection size, ranging from 0 to 5000, while the vertical axis represents the set size, spanning from 0 to 4000. The numerical sequences within the plot meticulously detail the intersection counts between each pair of sets, providing a clear and intuitive comparison of the overlap among different datasets. **(c)** This dotplot illustrates the enrichment of specialized genes in different Gene Ontology (GO) pathways within CD14 monocytes during the KD early group. The x-axis represents the gene ratio, while the y-axis lists the GO pathways. The size of the dots corresponds to the number of genes, and the color intensity indicates the level of statistical significance, with red signifying a more pronounced enrichment. **(d)** The expression difference plot of the ITK gene across various groups reveals that ITK is specifically expressed in the KD early group.

The control group had one module with 33 genes related to TH1/TH2 cell differentiation. The early vasculitis group had four modules, including one with 44 genes involved in T cell differentiation and receptor activation. The late vasculitis group had two modules (207 and 242 genes) with non-specific functions related to translation and ribosomal assembly. Notably, an UpSet analysis revealed extreme specificity of the ITK gene in the early vasculitis stage, highlighting its potential role in disease initiation (**Figures 7a–d**, **Supplementary Figure 6**, **Supplementary Tables 7**, **8**).

#### 3.3.5 CD16^+^ monocytes

The control group had seven modules, with functions related to cell adhesion and T cell receptor activation. The early vasculitis group had three modules, including one with 39 genes in the T cell receptor pathway and one with 348 genes in innate immune activation. The late vasculitis group had two non-specific modules (44 and 124 genes) related to translation and ribosomal function. These findings underscore the dynamic reprogramming of monocytes during disease progression (**Supplementary Figure 7**, **Supplementary Tables 9**, **10**).

## 4 Discussions

Kawasaki Disease (KD) is a systemic vasculitis that predominantly affects children, with the most significant impact on the coronary arteries. Although the etiology of KD remains incompletely understood, it is widely accepted that its pathogenesis involves the interplay between genetic susceptibility and environmental triggering factors (2). In the pathogenesis of KD, communication among immune cells plays a crucial role, particularly in the development of coronary artery damage. Studies have indicated that in KD, immune cells such as T cells, B cells, macrophages, and neutrophils communicate with each other by secreting cytokines and expressing surface molecules, initiating inflammatory responses and vascular damage (7, 18). Notably, the differentiation of CD8^+^ T cells is impaired in KD, which may affect the repair and regenerative capacity of the coronary arteries (6). Immune cell communication is not limited to within the immune cell community; it also plays a key role in diseases with coronary artery damage. Certain signaling molecules, by binding to their ligands, modulate antigen presentation and the activation status of immune cells, influencing T and B cell immune responses, promoting leukocyte recruitment and activation, which may lead to inflammation and damage in the coronary arteries. Moreover, these communication processes may also affect the function of vascular endothelial cells, including adhesion, migration, and vascular permeability, changes that could promote the development of coronary artery damage (19). In some vasculitis, the auto-communication of immune cells and their interactions with endothelial cells are particularly important (20). Intravenous immunoglobulin (IVIG) is the standard treatment for KD, but its precise mechanism of action is not fully elucidated. IVIG may exert its effects by modulating immune cell communication and inflammatory responses (18). In the treatment of other vasculitis, modulating the interactions between T cells and macrophages can also mitigate inflammation and vascular damage (21). Single-cell sequencing technology has been employed to analyze the immune cell communication in the acute phase of KD at the transcriptome level, aiming to gain a deeper understanding of the immune communication characteristics of KD at different stages of the disease course and to explore key targets within these processes.

In the study findings, an increased quantity of CD14^+^ monocytes was noted during the early phases of vasculitis, which were also the primary sources of significant signaling. In contrast, during the later stages of vasculitis, the count of CD14^+^ monocytes diminished, even falling below the levels observed in healthy states. Recent literature has reported a marked down regulation of CD14 on monocytes in the acute phase of Kawasaki Disease (KD), with therapeutic responses to intravenous immunoglobulin (IVIG) and infliximab correlating with the restoration of CD14 expression on monocytes (22). Some studies have also documented an elevation in CD14 expression during the acute phase of KD (23). The research highlights the dynamic shifts in cell counts at various junctures of the vasculitis process. CD14, serving as a central receptor for pattern recognition receptors such as Toll-like receptors (TLRs), engages with TLR1/TLR2, TLR2/TLR6, TLR4, and TLR9 to identify and bind a spectrum of pathogen-associated molecular patterns (PAMPs) and damage-associated molecular patterns (DAMPs). The engagement of CD14 with PAMPs and DAMPs promotes the internalization of CD14-TLR complexes, resulting in a down regulation of CD14 expression on monocytes and the initiation of inflammatory responses (24). This sequence of events may persist from the onset to the later phases of KD vasculitis, accounting for the observed biphasic trend of CD14^+^ monocyte numbers.

In the early stages of vasculitis, CD14^+^ monocytes, known for their highest signal transduction, act as a central hub in cellular communication network, with SELPLG gene expression being the most intense among all signals analyzed. The SELPLG gene encodes for the selectin P ligand 1 (PSGL-1), a cell surface molecule predominantly found on leukocytes. PSGL-1 interacts with P-selectin present on platelets and activated endothelial cells, playing a crucial role in leukocyte rolling and adhesion processes (25). In the context of coronary artery disease, polymorphisms within the SELPLG gene may correlate with fluctuations in plasma SELPLG levels, potentially impacting PSGL-1 expression or its binding affinity to P-selectin, and thus influencing disease progression (26).Within the co-expression module analysis, the ITK gene was notably distinctive in CD14^+^ monocytes during the early phases of vasculitis. ITK, a T-cell co-stimulatory gene, is integral to T-cell activation, which requires two signals: the first from the T-cell receptor (TCR) binding to antigen peptides presented by major histocompatibility complex (MHC) molecules, and the second from the interaction between co-stimulatory molecules on T-cells and their ligands on antigen-presenting cells (APCs) (27). Beyond the active signaling of MHC class I molecules, these co-stimulatory genes work in concert to activate CD8^+^ T lymphocytes, reinforcing the concept of CD14^+^ monocytes as communication hubs in early vasculitis. ITK plays a pivotal role in TCR signaling, influencing the release of pro-inflammatory cytokines by T cells, and is linked to the pathogenesis of various autoimmune diseases and tumors. Experimental models have demonstrated therapeutic effects with the absence or inhibition of ITK, particularly in conditions such as asthma and inflammatory bowel disease (28). In Kawasaki Disease, these two genes are instrumental in the activation of CD8^+^ T-cells by CD14^+^ monocytes, highlighting their significance in disease pathology.

CD8^+^ T lymphocytes are the recipients of all initiating signals and instrumental in the immune response, tasked with the critical role of eliminating cells infected by viruses, neoplastic cells, and those abnormally activated in autoimmune conditions (29). In the context of Kawasaki Disease (KD), the dynamics of CD8^+^ T cells, in terms of both function and quantity, are subject to alteration. Research has demonstrated that during the acute phase of KD, CD8^+^ T cells are hyperactivated, as evidenced by an elevated proportion of CD8^+^HLA-DR^+^ T cells (30, 31). The current study reveals an augmented interaction between CD14^+^ monocytes and CD8^+^ T cells in the early stages of vasculitis, coincident with an up regulation of HLA molecules on CD8^+^ T lymphocytes. This finding implies that CD14^+^ monocytes may predominantly activate CD8^+^ T lymphocytes during the initial phase of vasculitis. Certain subsets of CD8^+^ T cells in KD, such as T follicular helper (Tfh) cells, have been implicated in the pathogenesis of coronary artery aneurysms (32, 33). The activation status and numerical changes in CD8^+^ T cells offer a diagnostic window into KD and a predictive measure of patient responsiveness to IVIG therapy. Aberrant activation of CD8^+^ T cells in KD could potentially underlie resistance to IVIG, and thus, dampening this over activation may enhance treatment efficacy (34, 35). Consequently, the findings suggest that targeting the activation of CD8^+^ T cells by CD14^+^ monocytes may serve as a therapeutic strategy in the early stages of KD vasculitis.

The investigation unveiled that throughout all observed time frames, the MHC-I genes were the most intensely signaled, a finding consistent with their established functions in antigen presentation, T cell activation, and the initiation of adaptive immune responses (36). The MHC region, known for its repertoire of immune-related genes, suggests that polymorphisms within these genes could modulate an individual’s susceptibility to Kawasaki Disease (37). The MHC molecules’ expression on vascular endothelial cells may also be implicated in the vasculitic processes of KD and associated with coronary artery lesions (38). Interestingly, within the KD cohort, MHC signaling molecules were markedly reduced compared to controls, while there was a notable increase in inflammatory cytokines such as IL-1, TNF, and CCL. These fluctuations in signaling molecule intensity can alter the vigor and nature of immune responses, thereby impacting the trajectory of KD. A comparative analysis of signal intensity between the early and late stages of vasculitis revealed disparities in CD40 gene activity. CD40, a tumor necrosis factor receptor superfamily member, is predominantly expressed on immune cells including B cells, T cells, and dendritic cells, as well as on non-immune cells like endothelial cells. It plays an essential role in adaptive immunity, particularly in the proliferation and differentiation of B cells (39). Interactions between CD40 and its ligand CD40L are crucial for communication between T and B cells, initiating and modulating immune responses (40, 41). Single nucleotide polymorphisms within the CD40 gene have been linked to an increased susceptibility to KD (42). Genetic variants in the CD40 gene may influence the expression or function of the CD40 protein, thus affecting an individual’s predisposition to KD. During the acute phase of KD, elevated CD40 expression may enhance immune cell activation and intensify inflammatory responses (43), potentially contributing to the development of coronary artery lesions (CALs). The CD40-CD40L interaction may also be instrumental in KD-associated inflammation and vascular damage (44), leading to endothelial cell activation and vascular inflammation that promote the formation of CALs. In this study, CD40 signaling was significantly different between the early and late stages of vasculitis, possibly due to the increasing communication between CD19^+^ B lymphocytes and CD8^+^ T cells, with a corresponding rise in CD40 signaling. Unlike its role in initiating vasculitis, CD40 appears to play a complex role in the perpetuation of vasculitis, involving immune cell activation, inflammation, and vascular damage, offering novel insights for the prediction of high-risk factors in KD.

This study leverages single-cell sequencing technology to reveal the pivotal role of CD14^+^ monocytes in the activation of vasculitis in Kawasaki Disease (KD), as well as the function of SELPLG and ITK as key signaling genes in cellular communication. These findings offer a new perspective on the immunopathological mechanisms of KD. In particular, targeting the communication pathways between CD14^+^ monocytes and CD8^+^ T cells may potentially improve the therapeutic outcomes of KD in the future. However, our study has several limitations. The limited sample size restricts the generalizability of our conclusions. Additionally, the lack of longitudinal single-cell sequencing data from the same patients over the course of the disease means that our study cannot fully capture the continuous dynamic changes of the disease. This limitation also restricts our ability to comprehensively understand the changes in cellular characteristics during disease progression. Moreover, the absence of mechanistic validation further underscores the need for additional evidence. Moving forward, we plan to expand the sample size to verify the robustness of our findings and conduct functional validations of the key signaling pathways through *in vitro* cellular experiments and animal models. We will also further explore the interplay between environmental and infectious factors and genetic susceptibility, providing a more comprehensive theoretical basis for the precise diagnosis and treatment of KD.

## 5 Conclusion

The application of single-cell sequencing technology has provided a novel approach and perspective for discovering different diagnostic biomarkers throughout the course of KD. CD14^+^ monocytes play a pivotal role in cellular communication during the activation process of KD vasculitis, with SELPLG and ITK being important communication signal genes.

## Data Availability

The datasets presented in this study can be found in online repositories. The names of the repository/repositories and accession number(s) can be found in the article/**Supplementary Material**.

## Supplementary material

Supplementary Figure 1 The PCA analysis of 10 samples based on the average expression value for each gene among all cells within one sample. By doing so, scRNA-Seq data could be utilized as pseudo-bulk RNA-Seq data. Top 5000 differentially expressed genes among 10 samples are select for PCA analysis in order to find the most significant variance among different samples.

Supplementary Figure 2 Clustering based on functional similarity, each dataset with similar functions is clustered separately, with different colors representing different functional clustering modules of genes.

Supplementary Figure 3 The soft threshold power for the control group meta-cohort was 6, for the early vasculitis group meta-cohort was 5, and for the late vasculitis group meta-cohort was 6. The height of the dendrogram represents the merging distance between different clusters, while different colors are used to distinguish between various cluster modules.

Supplementary Figure 4 The soft threshold power for the control group meta-cohort was 4, for the early vasculitis group meta-cohort was 2, and for the late vasculitis group meta-cohort was 2. The height of the dendrogram represents the merging distance between different clusters, while different colors are used to distinguish between various cluster modules.

Supplementary Figure 5 The soft threshold power for the control group meta-cohort was 14, for the early vasculitis group meta-cohort was 9, and for the late vasculitis group meta-cohort was 7. The height of the dendrogram represents the merging distance between different clusters, while different colors are used to distinguish between various cluster modules.

Supplementary Figure 6 The soft threshold power for the control group meta-cohort was 11, for the early vasculitis group meta-cohort was 15, and for the late vasculitis group meta-cohort was 8. The height of the dendrogram represents the merging distance between different clusters, while different colors are used to distinguish between various cluster modules.

Supplementary Figure 7 The soft threshold power for the control group meta-cohort was 4, for the early vasculitis group meta-cohort was 2, and for the late vasculitis group meta-cohort was 4. The height of the dendrogram represents the merging distance between different clusters, while different colors are used to distinguish between various cluster modules.

Supplementary Table 1 Detailed Gene Functions of Co-expression Modules in B Cells of Three Groups Identified by WGCNA.

Supplementary Table 2 Identification of Unique Element Genes in Different Expression Modules of B Cells by UpSet Analysis.

Supplementary Table 3 Detailed Gene Functions of Co-expression Modules in CD4^+^T Cells of Three Groups Identified by WGCNA.

Supplementary Table 4 Identification of Unique Element Genes in Different Expression Modules of CD4^+^T Cells by UpSet Analysis.

Supplementary Table 5 Detailed Gene Functions of Co-expression Modules in CD8^+^T Cells of Three Groups Identified by WGCNA.

Supplementary Table 6 Identification of Unique Element Genes in Different Expression Modules of CD8^+^T Cells by UpSet Analysis.

Supplementary Table 7 Detailed Gene Functions of Co-expression Modules in CD14^+^ monocytes Cells of Three Groups Identified by WGCNA.

Supplementary Table 8 Identification of Unique Element Genes in Different Expression Modules of CD14^+^monocytes Cells by UpSet Analysis.

Supplementary Table 9 Detailed Gene Functions of Co-expression Modules in CD16^+^ monocytes Cells of Three Groups Identified by WGCNA.

Supplementary Table 10 Identification of Unique Element Genes in Different Expression Modules of CD16^+^T Cells by UpSet Analysis.
