# Supplementary figures and images for: CD14+ monocytes: the immune communication hub in early vasculitis symptoms of Kawasaki disease
Source: Front Immunol. 2025 Mar 26;16:1557231. doi: 10.3389/fimmu.2025.1557231 (PMC11979218; doi:10.3389/fimmu.2025.1557231)

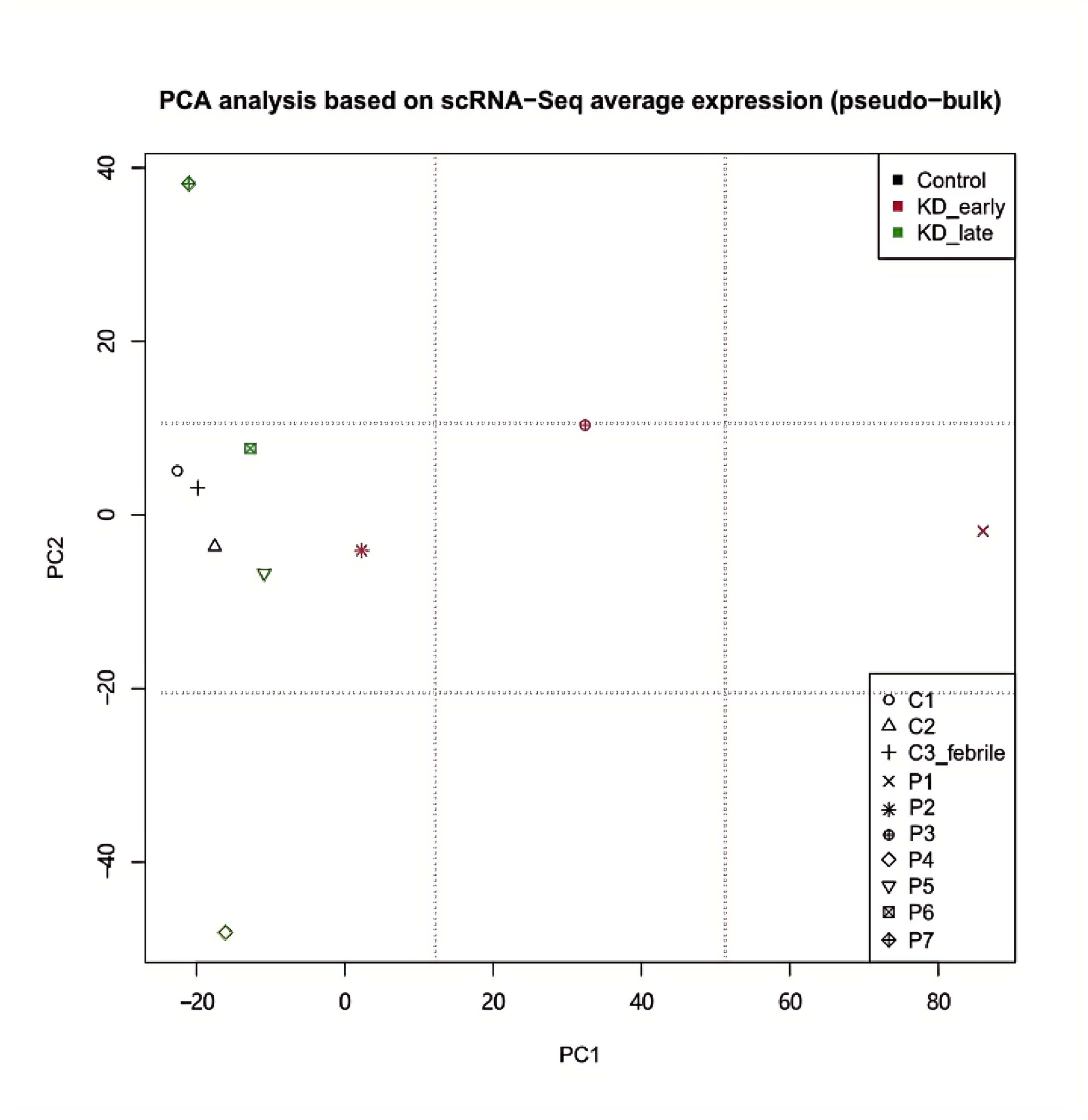

Supplement: Supplementary Figure 1 — The PCA analysis of 10 samples based on the average expression value for each gene among all cells within one sample. By doing so, scRNA-Seq data could be utilized as pseudo-bulk RNA-Seq data. Top 5000 differentially expressed genes among 10 samples are select for PCA analysis in order to find the most significant variance among different samples. [file Image1.jpeg]

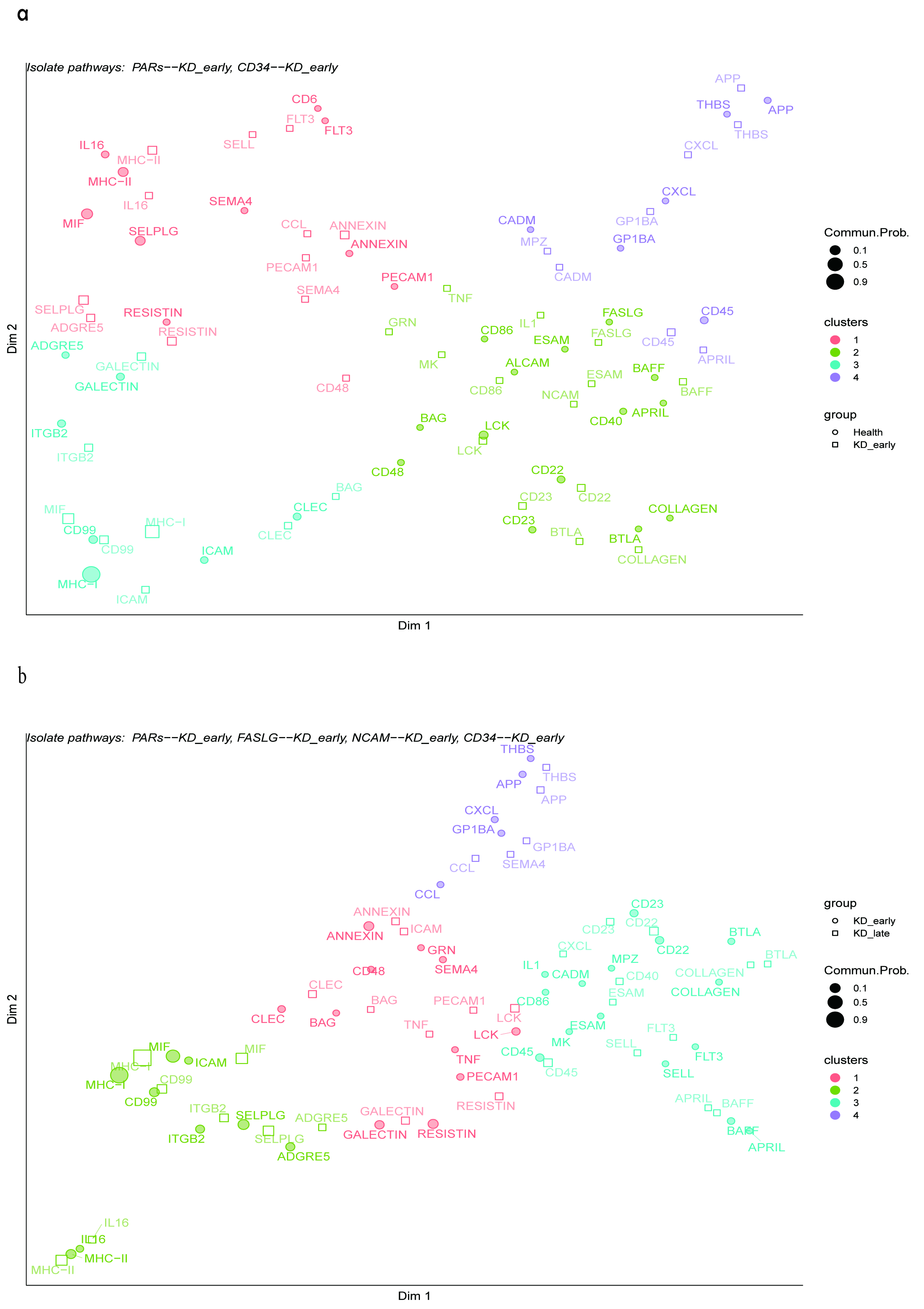

Supplement: Supplementary Figure 2 — Clustering based on functional similarity, each dataset with similar functions is clustered separately, with different colors representing different functional clustering modules of genes. [file Image2.tif]

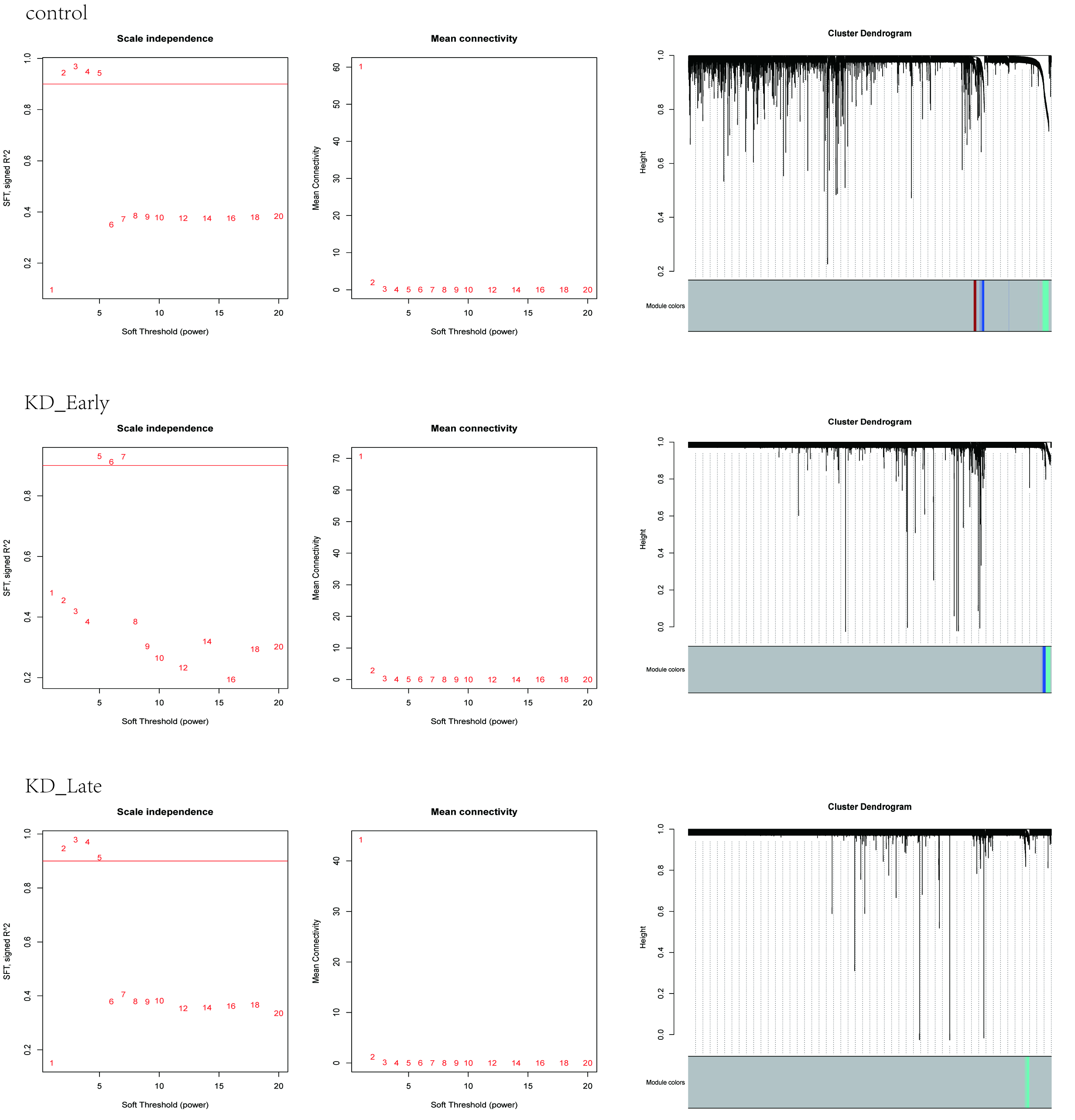

Supplement: Supplementary Figure 3 — The soft threshold power for the control group meta-cohort was 6, for the early vasculitis group meta-cohort was 5, and for the late vasculitis group meta-cohort was 6. The height of the dendrogram represents the merging distance between different clusters, while different colors are used to distinguish between various cluster modules. [file Image3.tif]

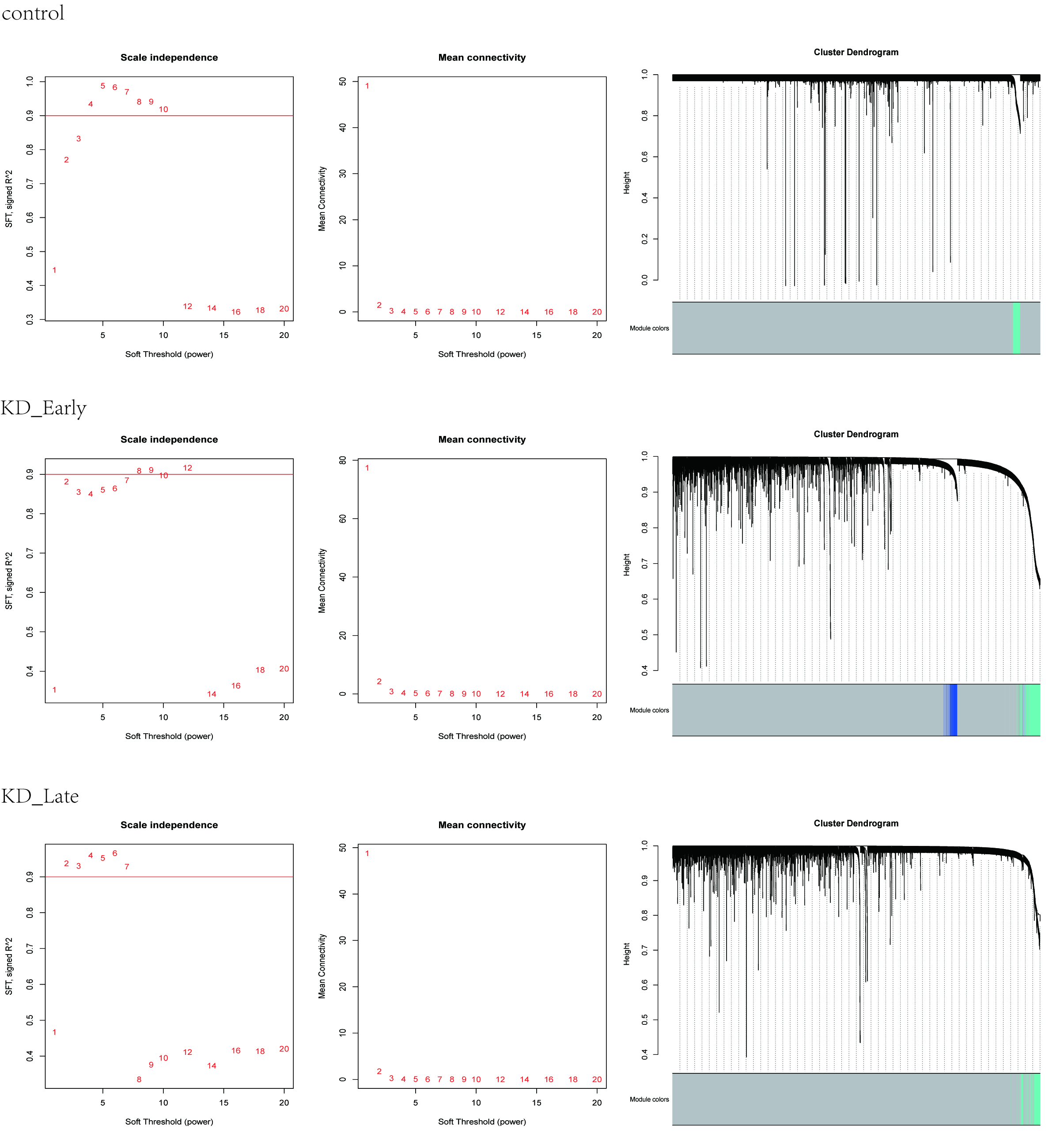

Supplement: Supplementary Figure 4 — The soft threshold power for the control group meta-cohort was 4, for the early vasculitis group meta-cohort was 2, and for the late vasculitis group meta-cohort was 2. The height of the dendrogram represents the merging distance between different clusters, while different colors are used to distinguish between various cluster modules. [file Image4.tif]

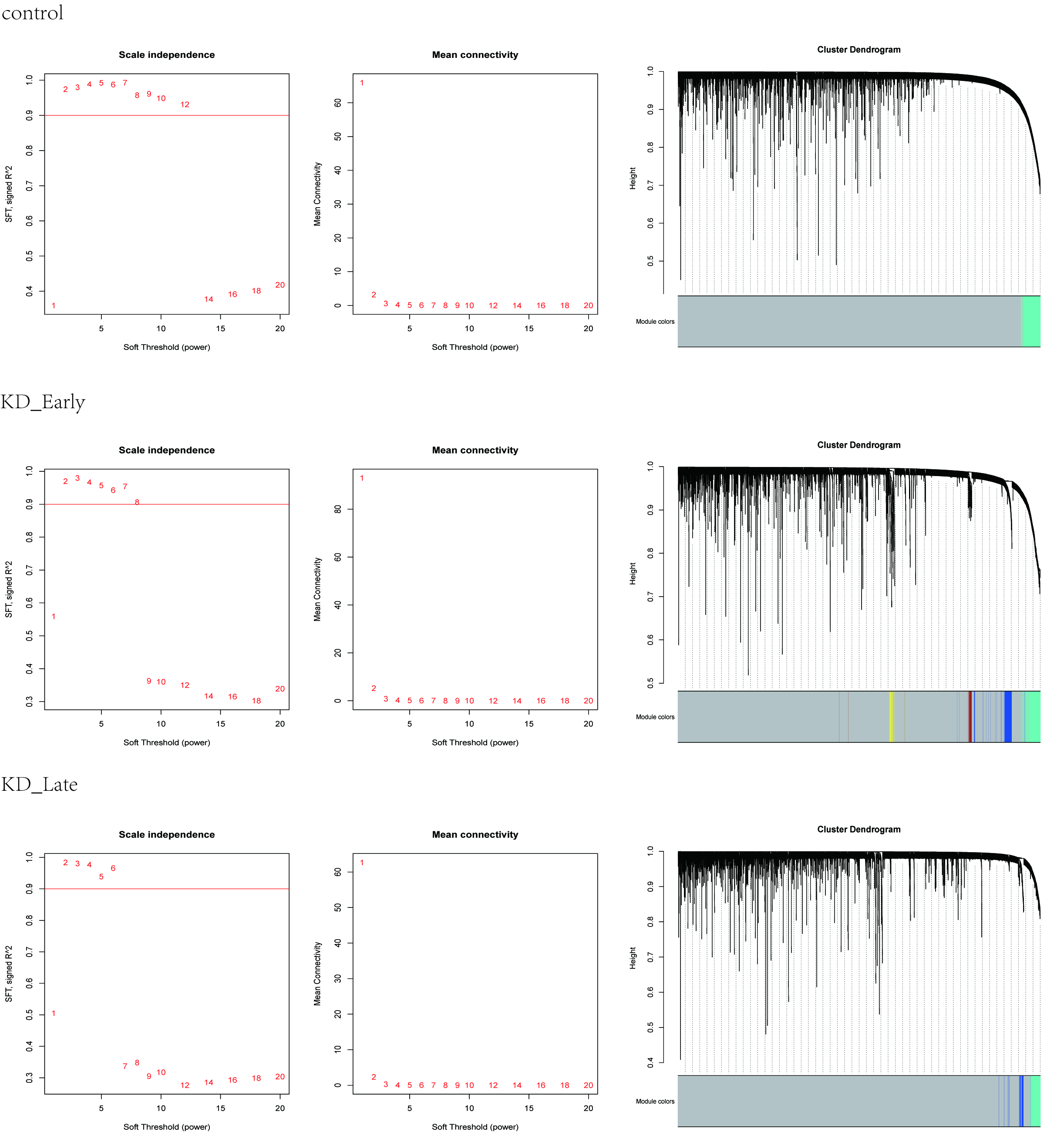

Supplement: Supplementary Figure 5 — The soft threshold power for the control group meta-cohort was 14, for the early vasculitis group meta-cohort was 9, and for the late vasculitis group meta-cohort was 7. The height of the dendrogram represents the merging distance between different clusters, while different colors are used to distinguish between various cluster modules. [file Image5.tif]

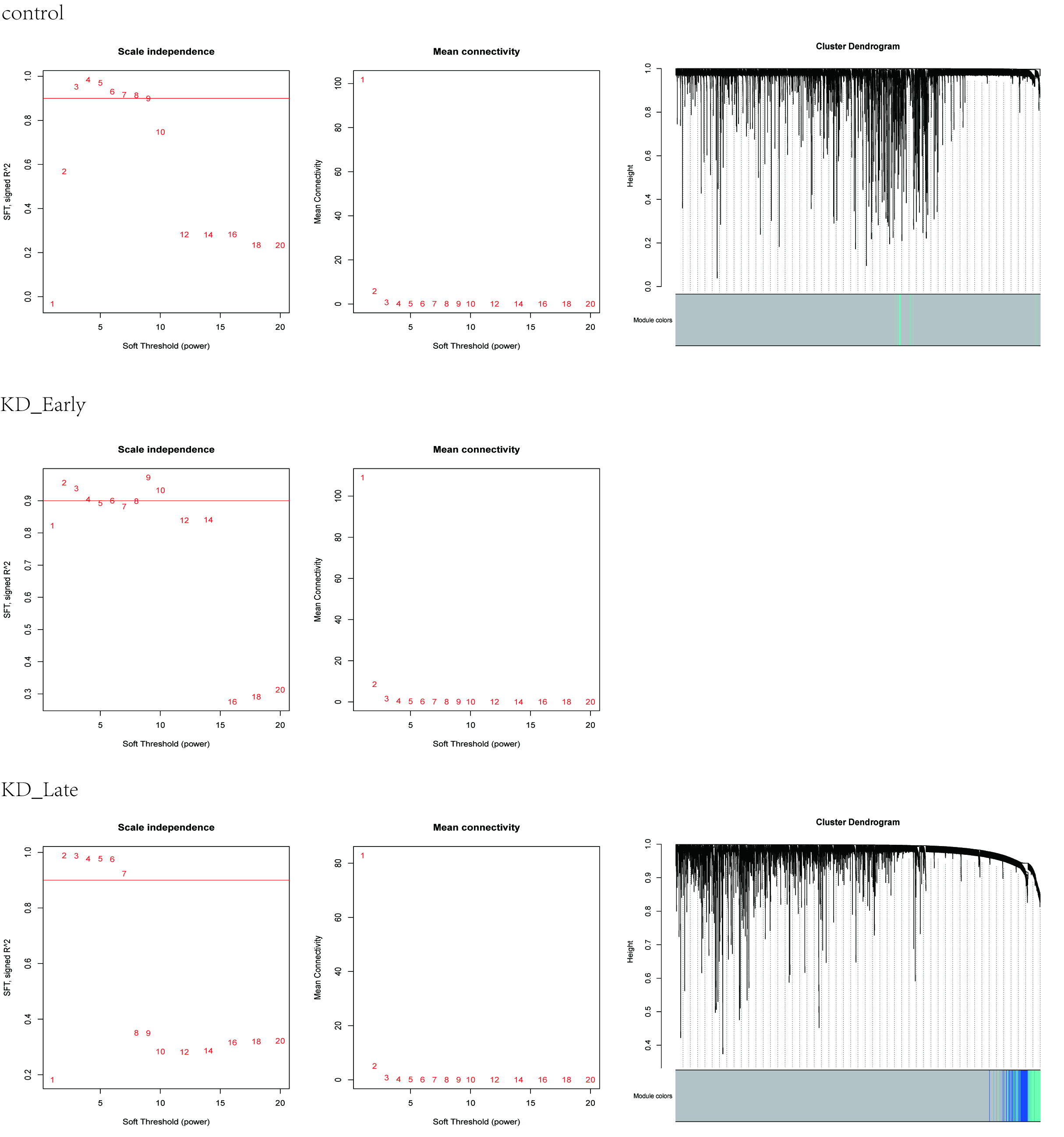

Supplement: Supplementary Figure 6 — The soft threshold power for the control group meta-cohort was 11, for the early vasculitis group meta-cohort was 15, and for the late vasculitis group meta-cohort was 8. The height of the dendrogram represents the merging distance between different clusters, while different colors are used to distinguish between various cluster modules. [file Image6.tif]

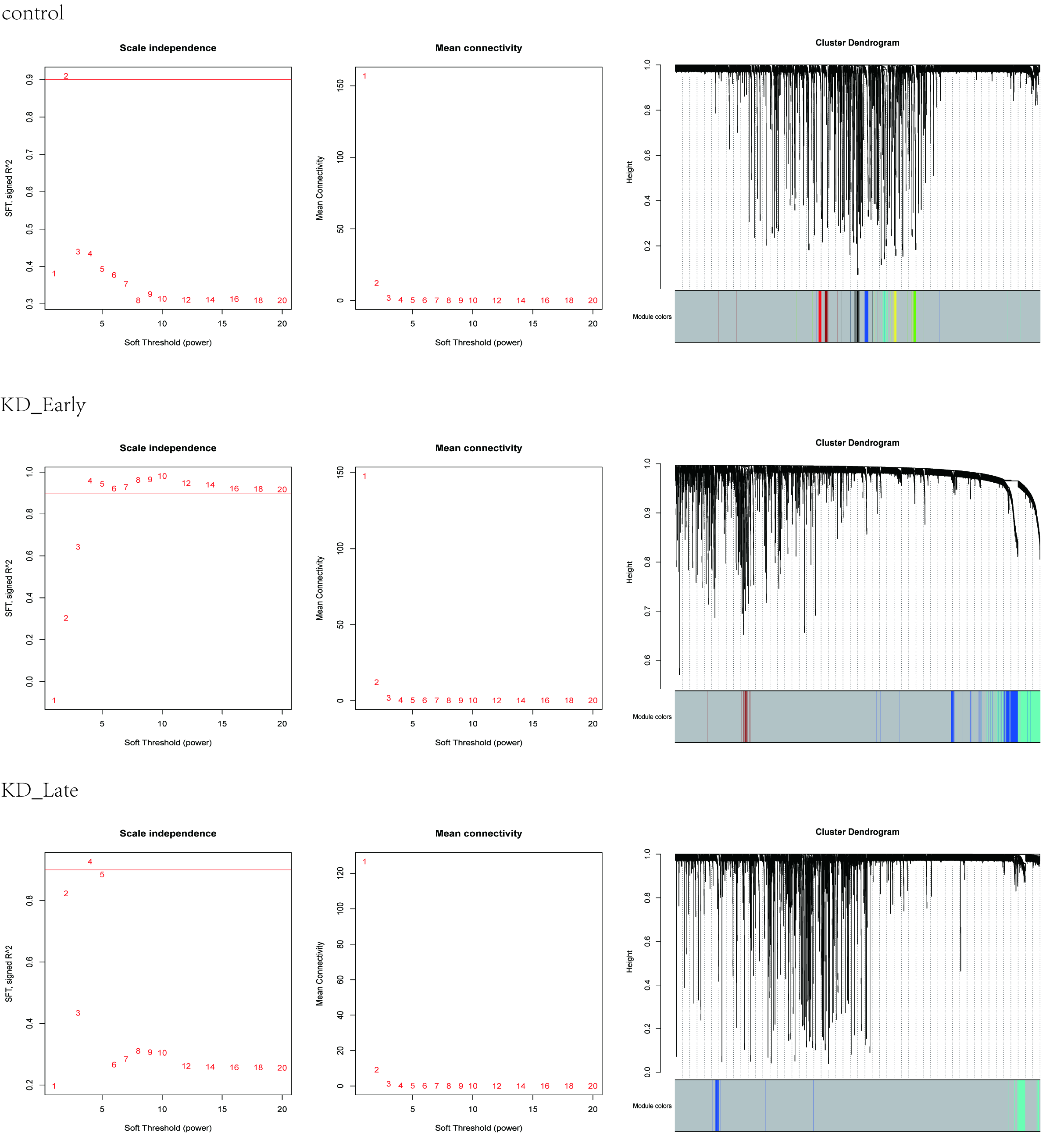

Supplement: Supplementary Figure 7 — The soft threshold power for the control group meta-cohort was 4, for the early vasculitis group meta-cohort was 2, and for the late vasculitis group meta-cohort was 4. The height of the dendrogram represents the merging distance between different clusters, while different colors are used to distinguish between various cluster modules. [file Image7.tif]
